# Empirical assessment and comparison of neuro-evolutionary methods for the automatic off-line design of robot swarms

**DOI:** 10.1038/s41467-021-24642-3

**Published:** 2021-07-16

**Authors:** Ken Hasselmann, Antoine Ligot, Julian Ruddick, Mauro Birattari

**Affiliations:** IRIDIA, Université libre de Bruxelles, Brussels, Belgium

**Keywords:** Electrical and electronic engineering, Computer science

## Abstract

Neuro-evolution is an appealing approach to generating collective behaviors for robot swarms. In its typical application, known as off-line automatic design, the neural networks controlling the robots are optimized in simulation. It is understood that the so-called reality gap, the unavoidable differences between simulation and reality, typically causes neural network to be less effective on real robots than what is predicted by simulation. In this paper, we present an empirical study on the extent to which the reality gap impacts the most popular and advanced neuro-evolutionary methods for the off-line design of robot swarms. The results show that the neural networks produced by the methods under analysis performed well in simulation, but not in real-robot experiments. Further, the ranking that could be observed in simulation between the methods eventually disappeared. We find compelling evidence that real-robot experiments are needed to reliably assess the performance of neuro-evolutionary methods and that the robustness to the reality gap is the main issue to be addressed to advance the application of neuro-evolution to robot swarms.

## Abstract (web summary)

Off-line neuro-evolution produces robot swarms whose good performance in simulation does not often transfer to the real word. With an extensive empirical study, Hasselmann et al. substantiate overfitting as the dominant cause.

## Introduction

Neuro-evolutionary robotics^1^ is an appealing approach to realizing collective behaviors for robot swarms^2–4^. In this approach, each individual robot is controlled by a neural network that maps sensor readings to actuator commands. The parameters of the network, and possibly its topology, are obtained by optimizing a mission-specific performance measure via artificial evolution.

The neuro-evolutionary approach appears to be appropriate in swarm robotics^5^ because it bypasses the main problem that designers face: defining what the individual robots should do so that the desired collective behavior emerges from their interactions. This problem is particularly hard because of the complexity of the interactions between robots and the loosely-coupled nature of a robot swarm. Indeed, which robot interacts with which other and when this happens is unknown at design time, being the result of the contingencies experienced by the swarm at execution time. Although swarm robotics is considered as a prominent research direction^6–13^, no general approach has been proposed so far to defining what the individuals should do to obtain the desired collective behavior^3^—although approaches exist that solve the problem under specific hypotheses^14–22^. Because neuro-evolutionary robotics, likewise other more or less related optimization-based design methods^23–28^, bypasses the problem of explicitly reducing the desired collective behavior to the one of the individuals, it appears to be, together with the other optimization-based methods, the only truly general approach to realizing robot swarms.

Neuro-evolutionary methods for the design of collective behaviors for robot swarms can be divided in two classes: off-line and on-line design. In off-line design, the design process is performed based on computer simulation and the resulting control software is subsequently deployed to the robots. In on-line design, the design process is performed continuously while the swarm operates in the target environment. For further information on the off-line design of robot swarms and on its on-line counterpart, we refer the reader to Birattari et al.^29^, Bredeche et al.^30^, and to the references therein. For a review of the neuro-evolutionary approach to swarm robotics, we refer the reader to Francesca and Birattari^31^. We refer the reader also to the vast literature on neuro-evolutionary robotics applied to single- and multi-robot systems^32–35^.

In the literature on neuro-evolutionary swarm robotics, empirical assessments and comparative analyses are rare^31^. In particular, to the best of our knowledge, no study has been published that compares any neuro-evolutionary method on multiple missions and reports results obtained in experiments performed with real robots. Yet, there is a general understanding that, due to the so-called reality gap^36,37^—that is, the unavoidable difference between simulation models and the real world—results obtained in simulation cannot be considered as a valid assessment of a neuro-evolutionary method for the automatic off-line design of robot swarms. It has been conjectured that the reality-gap problem is a sort of “overfitting” of the conditions experienced during the design process, which takes place in simulation; as a consequence of this overfitting, the control software fails to generalize to reality^26,38–40^. Indeed, a performance drop when moving from simulation to reality has been often reported in the literature^26,39,41^. Some recent results indicate that the reality gap is a relative problem with some design methods that are affected to a great extent, while others appear to be intrinsically more robust^40^.

**Fig. 1:**
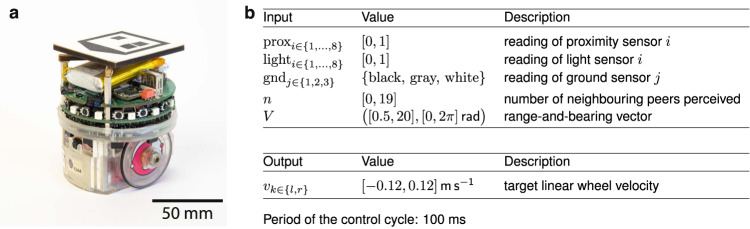
The robot and its reference model. **a** The e-puck robot in the configuration used for the experiments presented in the paper. Details are provided in “Methods”. **b** The reference model RM 1.1, which formally describes the programming interface through which, in the experiments presented in the paper, the control software interacts with the underlying hardware. The range-and-bearing vector points to the aggregate position of the neighboring peers perceived; its magnitude increases with the number of neighboring peers perceived and decreases with their distance. Formally, $V=\mathop{\sum }\nolimits_{m = 1}^{n}(\frac{1}{1+{r}_{m}},\angle {b}_{m})$, where *r*_*m*_ and ∠*b*_*m*_ are range and bearing of neighbor *m*, respectively. If no neighboring peer is perceived, the vector points in front of the robot and has unitary magnitude; formally, *V* = (1, ∠0).

In this paper, we present the results of an empirical study in which we assessed and compared some of the most advanced neuro-evolutionary methods for the off-line design of robot swarms. Figure 1 provides an image of the robot used in the experiments and the reference model that describes the programming interface through which the control software interacts with the underlying hardware. The results indicate that all the neuro-evolutionary methods under analysis are affected by the reality gap. This was possibly to be expected because of the aforementioned performance drop that has been often observed when moving from simulation to reality. What was not necessarily to be expected, because it had not emerged in any previous research, is that the extent to which the neuro-evolutionary methods under analysis are affected by the reality gap is so conspicuous that all differences we observed in simulation disappeared in the real-robot experiments. Eventually, the control software they produced performed at most only marginally better than a trivial random walk behavior that we included in the study as a control.

## Results

### Experimental setup

The methods comprised in the study include: (a) Covariance Matrix Adaptation Evolutionary Strategy (CMA-ES)^42^, for generating both single- and multi-layer perceptrons. CMA-ES is widely considered as one of the most effective evolutionary algorithms available and is especially valued for its advanced search capabilities. (b) Exponential Natural Evolution Strategies (xNES)^43^, for generating, also in this case, both single- and multi-layer perceptrons. xNES is closely related to CMA-ES and is sometimes preferred to the latter because it is considered to be more principled, as all the update rules needed for covariance matrix adaptation are derived from a single mechanism. (c) Neuro-Evolution of Augmenting Topologies (NEAT)^44^, initialized with either a fully-connected single-layer perceptron or a network in which input and output nodes are disconnected; in both cases, we studied two sets of hyper-parameters, one that allows the generation of recurrent networks and one that does not. NEAT is particularly valued for its capability to shape the network topology automatically. (d) EvoStick^26^, a straightforward implementation of the most basic ideas of the neuro-evolutionary approach. To the best of our knowledge, EvoStick is the only neuro-evolutionary method for the automatic design of robot swarms that has been tested on more than a single mission without undergoing any manually-applied mission-specific adaptation^26,41^. EvoStick is without any doubt less sophisticated and advanced than its competitors CMA-ES, xNES, and NEAT.

As baselines, we included in the study also: (1) Chocolate^41^, a design method that belongs to the AutoMoDe family^26^. It generates control software by assembling predefined software modules into a probabilistic finite-state machine and by fine-tuning their free parameters. The software modules are low-level behaviors (e.g., random walk, photo-taxis, stop) and conditions to transition from one low-level behavior to another (e.g., the floor is black, only few peers are perceived in the neighborhood). The modules are written once and for all in a mission-agnostic way. AutoMoDe was explicitly conceived to be robust to the reality gap. The definition of the approach was inspired by the notion of bias/variance trade-off of machine learning^45^. The idea was to introduce bias in the design process by restricting the design space—that is, the space of the possible behaviors that can be produced. Indeed, the space of the finite-state machines that can be produced by Chocolate is smaller than the space of the neural networks that can be produced by a typical neuro-evolutionary method. This bias was introduced with the goal of reducing the variance and preventing that the control software produced overfits the features of the simulator that do not have a counterpart in the real world^38,40^. This is eventually deemed to increase the intrinsic robustness of the method to the reality gap. (2) RandomWalk, a trivial behavior in which robots move randomly in the environment. Contrary to all the other aforementioned methods comprised in the study, RandomWalk is not an optimization-based design method: no parameter/feature of the behavior is optimized. For a design method, being unable to improve over RandomWalk is to be considered as a major failure.

We tested the methods under analysis for their ability to generate control software for five missions, in a fully automatic way. The missions were formally specified via a performance measure to be maximized, and the methods under analysis were tested on them without undergoing any manually-applied mission-specific modification. The control software generated by the methods was automatically cross-compiled for the target platform and was deployed without undergoing any modification. All the methods designed software for the same target platform, used the same realistic physics-based simulator with the same simulation models, and were provided the same resources—notably, the same number of simulation runs to be performed within the design process. Also, all the methods adopted the same devices that are widely considered as the standard practice for reducing the impact of the reality gap and for increasing the robustness of the control software generated: the injection of noise in simulation models and the randomization of the initial conditions^37^.

**Fig. 2:**
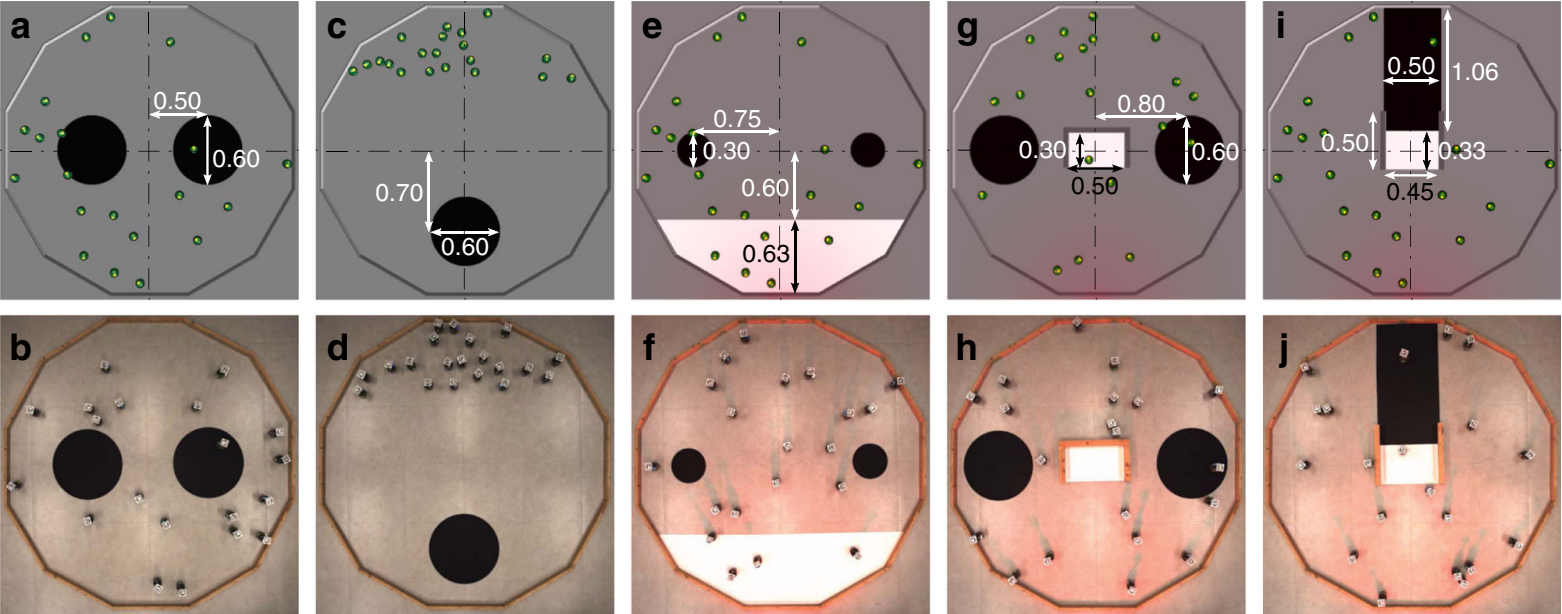
Arenas for the five missions. **a** XOR-AGGREGATION, simulation; **b**, real robots. **c**, HOMING, simulation; **d**, real robots. **e**, FORAGING, simulation; **f**, real robots. **g**, SHELTER, simulation; **h**, real robots. **i**, DIRECTIONAL-GATE, simulation; **j**, real robots. The 20 robots operate in a dodecagonal arena of 4.91 m^2^, the red glow in (**e**, **f**, **g**, **h**, **i**), and (**j**) indicates the presence of a light source at the bottom side of the arena. Dimensions (in meters) of the elements present in the arenas are given in (**a**, **c**, **e**, **g**,) and (**i**). Details on the experimental setup are provided in “Methods” and videos of the robot experiments are available as Supplementary Movies 1–5.

The five missions considered (Fig. 2) are rather typical collective missions. Their level of complexity is comparable with the one of those that, at the moment of writing, are customarily studied in the automatic off-line design of robot swarm. Admittedly, relatively more complex missions have been considered in the semi-automatic design literature—e.g., see Ferrante et al.^46^. This is understandable: semi-automatic design provides for human intervention within the design process and allows the designer to tailor the optimization process to the single specific mission considered. This eventually enables one to tackle relatively more complex missions that are out of reach for fully automatic design, at least at the current state of development of the field. As we have previously observed^47^, in (fully) automatic design, the challenge does not lie much in the complexity of each single mission, but rather in the fact that the design method must be able to produce control software for different missions without undergoing any modification.

We ran each method under analysis ten times on each of the five missions and we tested the control software they generated in real-robot experiments and also in simulation, so as to assess the impact of the reality gap on the different methods. A detailed description of all the methods, the robotics platform, the simulator, the five missions, the experimental design, and the statistics adopted is given in “Methods”.

### The impact of the reality gap

**Fig. 3:**
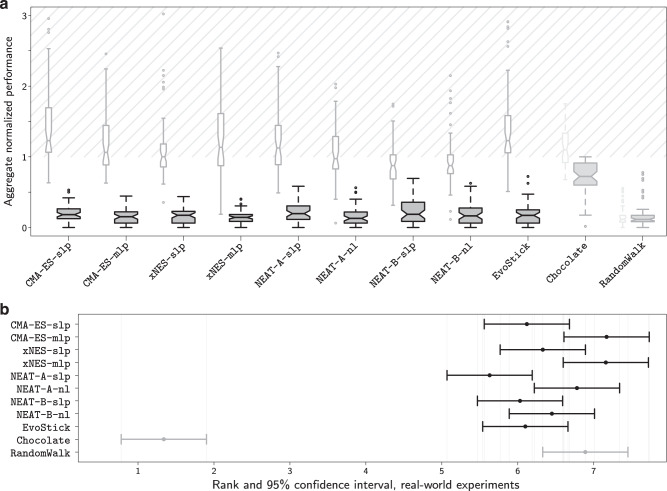
Aggregated results. **a** Aggregate performance in simulation (white narrow boxes) and in reality (gray wide boxes) across the five missions considered, represented by notched box-and-whisker plots, where the notches represent the 95% confidence interval on the median. If notches on different boxes do not overlap, the medians of the corresponding methods differ significantly, with a confidence of at least 95%. Graphical conventions adopted in box-and-whisker plots are described in “Methods” under the heading Statistics. Prior to the aggregation and for each missions, the results are normalized between the lowest and highest performance observed in reality by any of the design methods. As a result, the normalized performance in reality ranges between 0 and 1, but the one in simulation might exceed 1 (shadowed area). Indeed, in many cases, the performance observed in simulation exceeded the best one observed in the real-robot experiments. The performance of Chocolate and RandomWalk, the two methods included in the study as yardsticks, is grayed out so as to focus the attention of the reader to the neuro-evolutionary methods under analysis. **b** Friedman rank-sum test on the performance in reality: expected rank and 95% confidence interval. If two segments do not overlap, the rank of the corresponding methods differ significantly, with a confidence of at least 95%. Also here, the performance of Chocolate and RandomWalk is grayed out to focus the attention to the neuro-evolutionary methods. The videos of all robot experiments are available as Supplementary Movie 1–5.

**Fig. 4:**
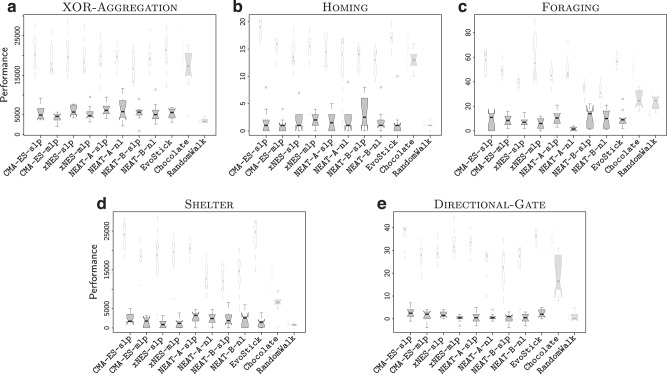
Results per missions. Performance obtained in simulation (white narrow boxes) and in reality (gray wide boxes) in all five missions: **a** XOR-AGGREGATION, **b** HOMING, **c** FORAGING, **d** SHELTER, **e** DIRECTIONAL-GATE. The results are presented using notched box-and-whiskers plots, where the notches represent the 95% confidence interval on the median. If notches on different boxes do not overlap, the medians of the corresponding methods differ significantly, with a confidence of at least 95%. Graphical conventions adopted in box-and-whisker plots are described in “Methods” under the heading Statistics. The performance of Chocolate and RandomWalk is grayed out so as to focus the attention of the reader to the neuro-evolutionary methods under analysis. The videos of all robot experiments are available as Supplementary Movie 1–5.

**Fig. 5:**
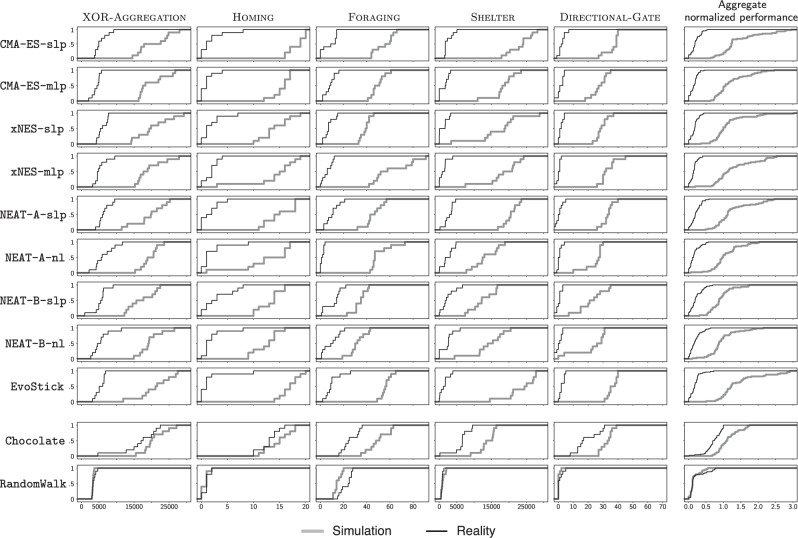
Distributions. Empirical distribution of the performance of the control software generated by each method under analysis on each of the five mission considered. The last column displays the normalized performance of each method, aggregated across the five missions. Aggregation is performed using the min-max normalization technique described in Methods under the heading Statistics. The black line represents the empirical distribution of the performance observed in real-robot experiments; the gray one, the one obtained in simulation.

The results (Figs. 3 and 4) show that, on the missions considered in the study, all the neuro-evolutionary methods under analysis experienced a major drop in performance because of the reality gap. For each mission and method, the empirical distributions of all the data gathered in simulation and real-robot experiments are given in Fig. 5.

**Fig. 6:**
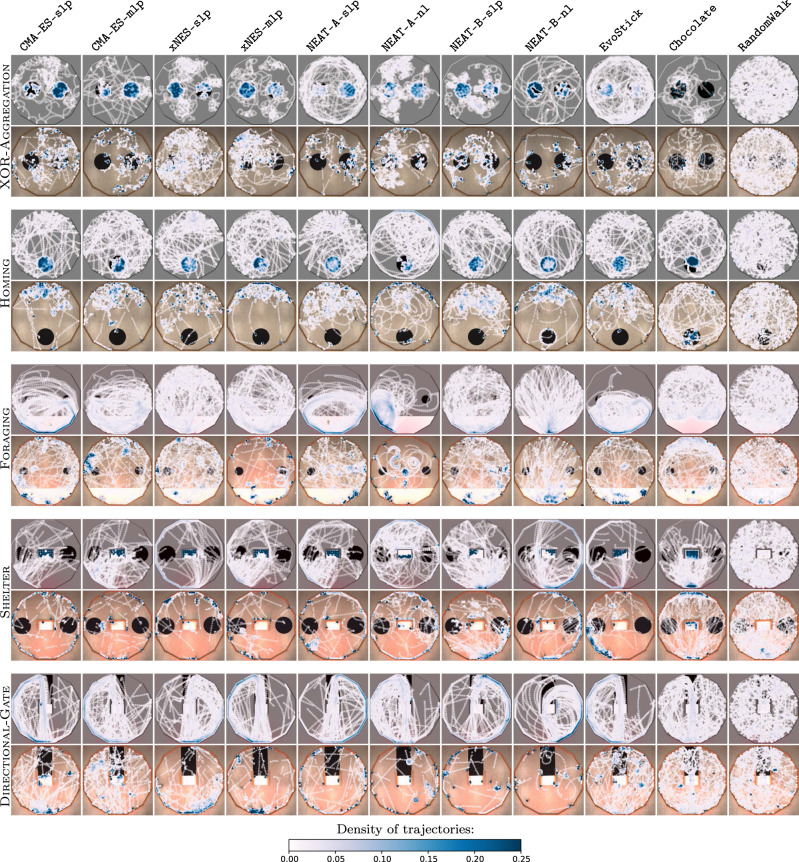
Trajectories of the robots throughout the entire median runs. For each method on each mission, we report the execution in simulation (top row for each mission) and reality (bottom row for each mission) of the instance of control software that obtained a median performance in reality, out of the instances produced by that method for that mission. The color of a spot represents the amount of time a robot spent on that spot during the execution. If a robot were to stay on a spot for more than a quarter of the entire execution, the color of that spot would be dark blue (value 0.25 in the color scale). The figure indicates that the control software produced by the evolutionary approaches cover the space differently in simulation and reality: in reality, the robots tend to form clusters, mostly against the walls. Differences between simulation and reality are less pronounced for Chocolate and barely noticeable for RandomWalk. For each mission and each method, a direct comparison of the behavior in simulation and reality is available in Supplementary Movie 6.

When evaluated in simulation, the control software produced by the neuro-evolutionary methods under analysis generally performed well, and comparably with the one of Chocolate; in some cases, even better. All methods under analysis performed significantly better than RandomWalk (Fig. 3a). Results were different when the control software was evaluated in real-robot experiments. The performance of all design methods dropped due to the reality gap, as it is often the case. Only the performance of RandomWalk remained substantially stable—this because, as observed above, RandomWalk is not a design method: no optimization process is involved and therefore overfitting does not happen. All the neuro-evolutionary methods experienced a large drop, whereas the one of Chocolate is relatively smaller. Also from a qualitative point of view, the control software produced by the neuro-evolutionary methods displayed different behaviors in simulation and reality, whereas the one produced by Chocolate behaved similarly in simulation and reality, and even more so RandomWalk—see Fig. 6 and Supplementary Movie 6. Eventually, all neuro-evolutionary methods performed significantly worse than Chocolate and their results were only marginally better than those of RandomWalk (Fig. 3a).

It is important to notice that the reality gap faced by the methods under analysis is the same: they all adopt the same simulator and design control software for the same platform. Yet, the extent to which the methods were affected is different. As it has been already observed^40^, the reality-gap problem is a relative problem, with some methods being more heavily affected and others being intrinsically more robust.

The simulator provides a reasonably faithful representation of reality. This is particularly clear if one compares the performance in simulation and reality of RandomWalk, which does not involve any optimization performed in simulation and is therefore not exposed to overfitting—see Supplementary Movie 6. This allows one to appraise the accuracy of the simulator, independently of any overfitting that, as mentioned above, varies on a per-method basis.

A further observation is that the five missions can be accomplished to a satisfactory extent under the experimental conditions considered. Specifically, they can be accomplished by the platform adopted and by a robot swarm of the given size. Moreover, control software to accomplish these missions can be produced automatically using the available resources: the simulator provides a reasonably faithful representation of reality (albeit not perfect, as no simulation does) and the number of simulation runs allotted to each design process was appropriate. This is shown by the satisfactory results obtained in the robot experiments by Chocolate—see Supplementary Movies 1–5. Concerning the neuro-evolutionary methods, the fact that the number of simulation runs allotted was sufficiently large is confirmed by the satisfactory performance obtained in simulation by the control software they produced.

## Discussion

The performance of the different neuro-evolutionary methods under analysis is similar. The few differences that can be observed between the results obtained in simulation disappeared when the control software generated was tested in real-robot experiments. A remarkable fact is that, on the missions considered, the more advanced methods—that is, CMA-ES, xNES, and NEAT—did not yield any relevant improvement over EvoStick, the straightforward implementation of the neuro-evolutionary approach. This holds true both for simulation and robot experiments. The data indicate that, at least on the missions considered, neither the effective search of CMA-ES and xNES, nor the advanced abilities of NEAT to shape the topology of networks have the potential to improve the performance of the neuro-evolutionary approach. The real issue to be addressed is the robustness to the reality gap.

In the missions considered, the main discrepancies between the behaviors observed in simulation and reality concern the way in which robots cover the space. Robots tend to cluster (mostly against the walls) in reality more than they do in simulation. This is likely due to the fact that friction between robots and between robots and walls is not modeled in a sufficiently accurate way: in simulation, robots slip against each other and against the walls; while in reality, they remain more easily stuck. Another discrepancy we observed concerns the shape of the trajectories. In simulation, all robots can be observed to move orderly, following circular trajectories; in reality, some robots display similar trajectories while those of others tend to be squashed and irregular. This is likely due to the fact that, although the swarm is in principle homogeneous and it is simulated as such, the real robots tend to differ slightly one from the other in their sensors and actuators. As a result, the real robots fail to display the ordered and cohesive collective motion that can be observed in simulation. The issue is particularly noticeable in FORAGING and DIRECTIONAL-GATE. Both discrepancies (clustering and irregular trajectories) are accrued by density: the more the robots converge to a same restricted area, the more the behavior observed in reality differs from the simulated one. Although the discrepancies observed can be used to improve the simulator for future applications, we do not think that they provide information that can contribute to address the reality-gap problem in a universally valid way. It should be noted that reducing the differences between simulation and reality on the basis of the observation of control software produced by specific methods on specific missions could lead to ad hoc solutions that do not necessarily generalize to other methods, missions, platforms, environment, and scenarios^32,35,48^. Also, reducing the differences between simulation and reality a posteriori—that is, after observing that the control software produced in simulation does not behave satisfactorily in reality—is not compatible with the spirit and purpose of automatic off-line design as it requires human intervention and assessments on real robots.

The satisfactory results obtained in the real-robot experiments by Chocolate—both in absolute terms and relatively to those obtained by the neuro-evolutionary methods under analysis—corroborate the validity of the original idea that motivated the definition of AutoMoDe and the development of Chocolate itself. Indeed, the results confirm that a restricted design space is associated with a reduced risk of overfitting and an increased robustness to the reality gap. It is our contention that, in the experiment presented above, Chocolate crossed the reality gap successfully because of its relatively small design space. By the same token, we contend that the neuro-evolutionary methods under analysis failed to cross the reality gap successfully because, in their definition, no explicit attention was made to restrict the size of the design space.

Supported by the results presented above, we contend that, to advance the application of neuro evolution to the automatic off-line design of collective behaviors for robot swarms, the research community should focus on addressing the reality-gap problem. A number of ideas have already been proposed in the literature and belong into two distinct approaches^40^: (1) increase the accuracy of simulators as much as possible; (2) conceive design methods that are intrinsically robust to the reality gap. The two approaches are not mutually exclusive and can profitably coexist within the same design method. We definitely agree that simulation accuracy must be pursued. Yet, as simulation models will never be perfect and the risk of overfitting cannot be eliminated altogether, the quest for accurate simulators does not eliminate the need for robust methods. It is therefore our contention that future research should aim at increasing the intrinsic robustness of design methods. Making the optimization algorithms more effective or enhancing their ability to automatically shape the topology of the networks appears to be a secondary concern, at least in this phase of the development of the field.

Although most of the ideas proposed to address the reality-gap problem do not fit the framework of the automatic off-line design considered here and, to the best of our knowledge, have never been applied in swarm robotics, they could be possibly adapted or could be the starting point to develop original methods for enhancing the robustness of neuro-evolutionary robotics. For example, Koos et al.^39^ proposed a method that builds and updates a model of the differences between the performance in simulation and reality. The model is used to constrain the design process to generate only control software whose real-world performance is expected to be correctly predicted by the simulator. The method requires periodic runs with the robots during the design process, which cannot therefore rely on simulation only. Floreano and Mondada^49^ proposed a method that originally blends ideas from off-line and on-line design: the update rules of the neurons and their parameters are defined off-line in simulation; the synaptic weights are subsequently adapted on-line. Also the idea underlying the development of Chocolate—that is, restricting the design space to reduce the risk of overfitting—could be possibly applied in the context of neuro-evolutionary robotics. Indeed, restricting the design space is effectively a form of regularization and a variety of regularization techniques that could be ported to neuro-evolutionary robotics have already been described in the neural network literature^50,51^. For example, although further research is needed to develop a reliable method, previous results^52^ indicate that a popular regularization technique known as early stopping^53–56^ has the potential to increase the robustness to the reality gap of neuro-evolutionary methods for the automatic design of robot swarms. In the light of the results of our experiments, we are convinced that the adoption of an appropriate regularization technique is the most promising direction to be explored in the development of the neuro-evolutionary approach to the automatic off-line design of robot swarms.

Our main conclusions are: (i) Experiments with real robots are of paramount importance to have a correct picture: simulation gives a falsely overoptimistic assessment of the methods under analysis. (ii) The advanced features of CMA-ES, xNES, and NEAT do not appear to provide any practical advantage over the straightforward EvoStick. In any case, possible (minor) differences observed between the methods in simulation disappear when the control software is ported to the robots. (iii) The real issue is the lack of robustness to the reality gap of the currently available neuro-evolutionary methods for the automatic off-line design of robot swarms. This is the issue on which, in our opinion, future research should focus. (iv) RandomWalk was substantially unaffected by the reality gap. This corroborates the conjecture that the performance drop associated with the reality gap is to be understood as the result of a sort of overfitting, which does not happen in the case of the random walk as it does not involve any optimization performed in simulation. (v) Chocolate experienced a much smaller performance drop than the neuro-evolutionary methods under analysis. This confirms the validity of the original idea of reducing the risk of overfitting by restricting the design space, which is effectively a regularization technique. It is our contention that this technique—or another of the several regularization techniques previously described in the neural network literature—can be ported to neuro-evolutionary robotics and is a promising avenue to address the reality-gap problem in the application of neuro-evolution to the automatic off-line design of control software for robot swarms.

## Methods

### e-puck

The robot used in the research is the e-puck^57^, a small differential-drive robot that measures 50 mm of height and 70 mm of diameter. The e-puck is equipped with several sensors and actuators including infrared transceivers to detect the presence of surrounding obstacles and/or measure the intensity of the ambient light, and ground sensors to read the gray-scale color of the ground beneath. For the purpose of the research presented here, the e-puck was enhanced with two extension boards: (i) the range-and-bearing^58^, which enables a robot to sense the presence of neighboring peers and estimate their relative position; and (ii) the Overo Gumstix, a Linux board that increases the computing power and flexibility of the robot. A picture of the e-puck in the configuration adopted in the experiments is given in Fig. 1a.

### Reference model

In this study, the e-puck is formally described by the reference model RM 1.1 given in Fig. 1b—see Hasselmann et al.^59^ for more details. All design methods comprised in the study generate control software that interacts with the e-puck exclusively through the variables defined in RM 1.1.

### Simulator

All simulations are performed using ARGoS^60^, a simulator specifically conceived to simulate robot swarms. We used version 48 of ARGoS, along with the ARGoS-Epuck library^61^, which provides models for all extension boards. The library also enables the cross-compilation of the control software for the e-puck platform so that it can be ported to the robots without any modification^62^. The models of the e-puck’s components have been conceived on the basis of real-world data sampled from the robot’s sensors and actuators, according to the best practice^37,63^.

### Arena

The robots operate in an arena of 4.91 m^2^ surrounded by walls and possibly containing obstacles. The floor is gray, with some regions that are white or black, depending on the mission to be performed—see below. In some missions, a single light source, placed next to the arena, is on for the whole duration of an experimental run. This light source is filtered with a red gel to avoid overexposure of the overhead camera of the tracking system (see Experimental setting), but still be detectable by the robots’ light sensors, which are particularly sensitive to the infra-red and the lower range of the visible spectrum.

### Design methods under analysis

All neuro-evolutionary methods under analysis generate neural networks with 2 output and 25 input nodes. The 2 outputs define the velocity of the wheels. Concerning the inputs, 1 is a bias node, 8 encode the readings of the proximity sensors, 8 those of the light sensors, 3 those of the ground sensors, 1 encodes the number of neighbors perceived, and 4 the projections of the range-and-bearing vector *V* on the four unit vectors that point at 45°, 135°, 225°, and 315° with respect to the head of the robot. Inputs and outputs are described by RM 1.1—see Fig. 1b. The values of the synaptic weights range in [ − 5; 5].

CMA-ES-slp is based on CMA-ES^42^, an evolutionary algorithm in which the population is described in statistical terms via the covariance matrix of its distribution—slp is the mnemonic for single-layer perceptron: the network generated has a fully-connected feed-forward topology without hidden layers. The population size *λ* and the initial step-size *σ*_0_ are hyper-parameters of the optimization algorithm. We set *λ* = 100, a common choice in the literature^31^; and *σ*_0_ = 5, that is, half the width of the parameter range, the initial population will therefore cover the entire search space—the same choice was made also in several other studies^64–66^.

CMA-ES-mlp is derived from CMA-ES-slp and differs from it only in the topology of the network, which is here a fully-connected feed-forward neural network with one hidden layer composed of 14 nodes, including a bias node. The size of the hidden layer is the average of the number of nodes in the input and output layers, as recommended by Heaton^67^—mlp is the mnemonic for multi-layer perceptron: the input nodes are initially all connected to the hidden nodes, which are in turn all connected to the output.

xNES-slp is based on xNES^43^, an evolutionary algorithm similar to CMA-ES but in which the update rule is defined in a principled way. The hyper-parameters and their values are the same as in CMA-ES-slp. Also the network topology is the same one adopted in CMA-ES-slp.

xNES-mlp is derived from xNES-slp and differs from it only in the network topology, which is here a fully-connected feed-forward neural network with one hidden layer of 14 nodes, including 1 bias node—the same topology adopted in CMA-ES-mlp.

NEAT-A-slp is based on NEAT^44^, a neuro-evolutionary algorithm that optimizes both the weights and the topology of the neural network. The design process is initialized with a fully connected feed-forward neural network with no hidden layers—slp is the mnemonic for single layer perceptron: the input nodes are initially all connected to the output. The hyper-parameters of NEAT-A-slp are those originally published by Stanley and Miikkulainen^44^ and recommended by them. They are labeled as pole2_markov in the original software package released by the authors.

NEAT-A-nl is derived from NEAT-A-slp and differs from it only in the initialization of the design process, which is here a disconnected network—nl is the mnemonic for no link: the input nodes are initially disconnected from the output.

NEAT-B-slp is similar to NEAT-A-slp and differs from it only in the value of a few hyper-parameters. The hyper-parameters of NEAT-B-slp are those labeled as params256 in the original software package published by Stanley and Miikkulainen^44^. The differences between set *A* (presented above) and *B* are that set *B* has a higher compatibility coefficient (leading to less species creation), set *A* penalizes old species whereas set *B* does not, and most importantly that set *B* can generate recurrent networks.

NEAT-B-nl is derived from NEAT-B-slp and differs from it only in the initialization of the design process, which is here a disconnected network: the input nodes are initially disconnected from the output.

EvoStick is a rather standard neuro-evolutionary robotics method. It was introduced by Francesca et al.^68^ and then used as a yardstick to evaluate other design methods^26,41^. To the best of our knowledge, EvoStick is the only neuro-evolutionary method that has been tested on more that one single mission without undergoing any mission-specific modification. EvoStick generates a fully-connected feed-forward neural network with no hidden nodes. EvoStick uses an evolutionary algorithm based on elitism and mutation. A population of 100 individuals is sampled at the beginning of the process; at each generation, the best 20 individuals are selected and passed unchanged to the following generation; random mutations are applied to these same 20 individuals to form the remaining 80 individuals of the new population.

Chocolate belongs to the AutoMoDe^26^ family of design methods. It generates control software by assembling predefined modules into probabilistic finite-state machines and by fine-tuning their free parameters^41^. The modules on which Chocolate operates are 6 low-level behaviors and 6 conditions. A low-level behavior is an action that a robot performs and a condition is a criterion for transitioning from the current low-level behavior to another one. The low-level behaviors are: exploration, stop, phototaxis, anti-phototaxis, attraction-to-neighbors, repulsion-from-neighbors. The 6 conditions are: black-floor, white-floor, gray-floor neighbor-count, inverted-neighbor-count, fixed-probability. The space of the possible combinations of the aforementioned modules is explored using Iterated F-race^69^.

RandomWalk is a ballistic-motion random walk: the robot moves straight until it encounters an obstacle. When this happens, the robot rotates on itself for a random number of timesteps and resumes it straight motion, if the path is clear; otherwise, it rotates for another random number of timesteps. This sequence is repeated indefinitely. RandomWalk is not an automatic design method as no parameter is tuned. It is included in the study as a lower bound on the performance.

### Missions

**XOR-AGGREGATION:** the robots must choose one of two black areas and aggregate to it. The size of the black areas and their positions are given in Fig. 2. The performance of the swarm is measured by the following objective function:

1 $${F}_{{\rm{a}}}=\mathop{\sum }\limits_{t=1}^{T}\mathop{\sum }\limits_{i=1}^{N}{I}_{i}(t);\qquad {I}_{i}(t)=\left\{\begin{array}{ll}1,&\,{\text{if}}\; {\text{robot}}\,\ i\ \,{\text{is}}\; {\text{in}}\; {\text{the}}\; {\text{area}}\; {\text{with}}\; {\text{the}}\; {\text{majority}}\; {\text{of}}\; {\text{robots}};\\ 0,&\,{\text{otherwise}}.\hfill\end{array}\right.$$

*T* = 180 s is the duration of the experimental run and *N* = 20 is the size of the swarm.

**HOMING:** the robots start in the upper part of the arena and must aggregate on the black area situated at the bottom. The size of the black area and its position are given in Fig. 2. The performance of the swarm is measured by the following objective function:

2 $${F}_{{\rm{h}}}=\mathop{\sum }\limits_{i=1}^{N}{I}_{i}(T);\qquad {I}_{i}(T)=\left\{\begin{array}{ll}1,&\,{\text{if}}\; {\text{robot}}\,\ i\ \,{\text{is}}\; {\text{in}}\; {\text{the}}\; {\text{black}}\; {\text{area}}\; {\text{at}}\; {\text{time}}\,T;\\ 0,&\,\text{otherwise.}\hfill\end{array}\right.$$

*T* = 120 s is the duration of the experimental run and *N* = 20 is the size of the swarm.

**FORAGING:** the robots must find one of the black areas, which represent food sources, and go back to the white one, which represents the nest. A light source is positioned behind the nest. The size of the areas of interest and their positions are given in Fig. 2. The performance of the swarm is measured by the following objective function:

3 $${F}_{{\rm{f}}}=K,$$

where *K* is the total number of round trips performed. The duration of an experimental run is *T* = 180 s and the swarm size is *N* = 20.

**SHELTER:** the robots must aggregate in the shelter, a rectangular white area positioned in the center of the arena and surrounded by walls on three sides. A light source is positioned outside the arena, in front of the open side of the shelter. The arena also features two black circular areas, next to the shelter. These areas do not have any predefined purpose/role in the definition of the mission: they are noise-features of the environment. The size of the shelter, the one of the black areas, and their positions are given in Fig. 2. The performance of the swarm is measured by the following objective function:

4 $${F}_{{\rm{s}}}=\mathop{\sum }\limits_{t=1}^{T}\mathop{\sum }\limits_{i=1}^{N}{I}_{i}(t);\qquad {I}_{i}(t)=\left\{\begin{array}{ll}1,&\,{\text{if}}\; {\text{robot}}\,\ i\ \,{\text{is}}\; {\text{in}}\; {\text{the}}\; {\text{shelter}};\,\\ 0,&\,{\text{otherwise}}.\hfill\end{array}\right.$$

*T* = 180 s is the duration of the experimental run and *N* = 20 is the size of the swarm.

**DIRECTIONAL-GATE:** the robots must traverse the gate, which is positioned in the center of the arena. They must do so from North to South. The gate is identified by white ground and the robots can follow a black corridor to reach it. The size of the gate, the one of the corridor, and their positions are given in Fig. 2. The performance of the swarm is measured by the following objective function:

5 $${F}_{{\rm{g}}}=K-K^{\prime} ,$$

where *K* is the number of times robots traverse the gate in the correct sense and $K^{\prime}$ is the number of times they traverse it in the wrong one. The duration of an experimental run is *T* = 120 s and the swarm size is *N* = 20.

### Protocol

#### Experimental setting

All real world experiments presented in this paper were performed in a controlled environment. The simulation environment was made to reproduce this setup as closely as possible. All experiments involved a swarm of 20 e-puck robots that operated in a wooden-wall arena with black and white paper patches on the ground. For each mission, each method was executed 10 times so as to obtain 10 instances of control software. Each design process was allowed the same budget of 200,000 simulation runs. To avoid introducing any bias, robot experiments were randomized and no experimental run performed was discarded. The performance of the swarm was computed automatically using data provided by a tracking system^70^ that registered the position of the robots throughout the duration of each experimental run. The position of the robots was not communicated to the robots themselves, which had only a local perception of the environment, coherently with the tenets of swarm robotics. The tracking system is based on an overhead camera and recognizes tags mounted on the robots—see Fig. 1a. Videos of all the experimental runs were recorded using the camera of the tracking system and are available as Supplementary Movies 1–5.

#### Statistics

We used notched box-and-whiskers plots to represent the performance of the different methods. In these plots, the thick horizontal line represents the median; the box extends to the upper and lower quartile; the upper/lower whiskers extends to the maximal/minimal observation that falls between the upper/lower quartile and 1.5 times the interquartile range; circles represent outliers, that is observations that fall beyond the whiskers. Notches on the box represent a 95% confidence interval on the median, and extend to $\pm 1.58\ \,\text{IQR}\,/\sqrt{n}$, where IQR is the interquartile range and *n* is the number of observations. The difference between the medians of two boxes is significant with a confidence of at least 95% if the notches of the respective boxes do not overlap^71^.

To aggregate the performances of the different methods across all missions, we used the min-max normalization technique: for each mission, we normalized the performances obtained in reality and in simulation with the minimal and maximal performance obtained in reality across all design methods. As a result, the normalized performance in reality ranges between 0 and 1, but the normalized performance in simulation might exceed 1 if instances of control software performed better in simulation than the maximal performance value obtained in reality. We also executed a Friedman test^72^, which aggregates all results by ranking the performance of all methods across all missions. We present the results in a plot that represents an estimate of the expected rank of each method and the relative 95% confidence interval. The performance of two methods is significantly different with confidence of at least 95% if the corresponding intervals do not overlap.

## Supplementary information

Peer Review File

Description of Additional Supplementary Files

Supplementary Movie 1

Supplementary Movie 2

Supplementary Movie 3

Supplementary Movie 4

Supplementary Movie 5

Supplementary Movie 6

Supplementary Data 1

## Data Availability

All results obtained in simulation and real robot experiments are available as Supplementary Data 1. Source data are provided with this paper.

## Source data

Source Data

## Code availability

The software used in the study is available online via the following repositories: ARGoS3-NEAT (10.5281/zenodo.4849517) for the implementation of NEAT-A-{slp∣nl}, NEAT-B-{slp∣nl} and EvoStick; ARGoS3-pagmo2 (10.5281/zenodo.4849533) for the implementation of CMA-ES and xNES; ARGoS3-AutoMoDe (10.5281/zenodo.4849541) for the implementation of Chocolate and RandomWalk; demiurge-epuck-dao (10.5281/zenodo.4849535) for the reference model of the robots used by all design methods; experiments-loop-functions (10.5281/zenodo.4849539) for the functions used to compute the score in the various missions; argos3-epuck (10.5281/zenodo.4882714) for the e-puck robot ARGoS3 plugin; ARGoS3 (10.5281/zenodo.4889111) for the ARGoS3 simulator; and irace (10.5281/zenodo.4888996) for the Iterated F-race algorithm. The scripts for installing the software and running the experiments and the control software automatically generated by the different methods comprised in the study presented here are available as Source Data.
