# Supplementary material for: Empirical assessment and comparison of neuro-evolutionary methods for the automatic off-line design of robot swarms
Source: Nat Commun. 2021 Jul 16;12:4345. doi: 10.1038/s41467-021-24642-3 (PMC8285396; doi:10.1038/s41467-021-24642-3)
Supplement: Supplementary file 2 — Description of Additional Supplementary Files [file 41467_2021_24642_MOESM2_ESM.pdf]

Description of Additional Supplementary Files

File Name: Supplementary Movie 1
Description: Aggregation XOR, real-robot experiment

File Name: Supplementary Movie 2
Description: Homing, real-robot experiment

File Name: Supplementary Movie 3
Description: Foraging, real-robot experiment

File Name: Supplementary Movie 4
Description: Shelter, real-robot experiment

File Name: Supplementary Movie 5
Description: Directional Gate, real-robot experiment

File Name: Supplementary Movie 6
Description: Summary of all experiments

File Name: Supplementary Data 1
Description: Raw data (.csv files) collected in simulations and real-robot experiments
