# Supplementary material for: Empirical assessment and comparison of neuro-evolutionary methods for the automatic off-line design of robot swarms
Source: Nat Commun. 2021 Jul 16;12:4345. doi: 10.1038/s41467-021-24642-3 (PMC8285396; doi:10.1038/s41467-021-24642-3)
Supplement: Supplementary file 1 — Peer Review File [file 41467_2021_24642_MOESM1_ESM.pdf]

Editorial Note: This manuscript has been previously reviewed at another journal that is not operating a transparent peer review scheme.
